# FastKnock: An efficient next-generation approach to identify all knockout strategies for strain optimization

**DOI:** 10.21203/rs.3.rs-3126389/v1

**Published:** 2023-07-10

**Authors:** Leila Hassani, Mohammad R. Moosavi, Payam Setoodeh, Habil Zare

**Affiliations:** Shiraz University; Shiraz University; Shiraz University; University of Texas Health Science Center

**Keywords:** genome-scale metabolic model, reaction knockout strategy, growth-coupled biosynthesis, biochemical overproduction, mathematical optimization, reaction clustering, search space reduction

## Abstract

Overproduction of desired native or nonnative biochemical(s) in (micro)organisms can be achieved through metabolic engineering. Appropriate rewiring of cell metabolism is performed making rational changes such as insertion, up-/down-regulation and knockout of genes and consequently metabolic reactions. Finding appropriate targets (including proper sets of reactions to be knocked out) for metabolic engineering to design optimal production strains has been the goal of a number of computational algorithms. We developed FastKnock, an efficient next-generation algorithm for identifying all possible knockout strategies for the growth-coupled overproduction of biochemical(s) of interest. We achieve this by developing a special depth-first traversal algorithm that allows us to prune the search space significantly. This leads to a drastic reduction in execution time. We evaluate the performance of the FastKnock algorithm using three *Escherichia coli* genome-scale metabolic models in different conditions (minimal and rich mediums) for the overproduction of a number of desired metabolites. FastKnock efficiently prunes the search space to less than 0.2% for quadruple and 0.02% for quintuple-reaction knockouts. Compared to the classic approaches such as OptKnock and the state-of-the-art techniques such as MCSEnumerator methods, FastKnock found many more useful and important practical solutions. The availability of all the solutions provides the opportunity to further characterize and select the most appropriate intervention strategy based on any desired evaluation index. Our implementation of the FastKnock method in Python is publicly available at https://github.com/leilahsn/FastKnock.

## Author summary

Metabolic systems biology aims to computationally analyze metabolic networks at systems level to comprehensively delineate their potential and probable functionalities. This approach is beneficial to metabolic engineers for finding appropriate rewiring targets and designing production strains with favorite metabolic behaviors. Recent in silico strain design approaches can identify several candidates and provide us with the opportunity of selecting the potentially operative solution among the identified candidates. Here, we present FastKnock, an efficient next-generation algorithm for effectual design of growth-coupled production strains. Among the existing algorithms, FastKnock is the only one capable of providing all possible solutions for multiple gene and reaction knockouts to overproduce a desired (bio)chemical. We achieve this by developing a special depth-first traversal algorithm that allows us to significantly prune the search space. The identification of all the solutions allows metabolic engineers to additionally evaluate, rank, and choose the most appropriate intervention strategies. FastKnock’s users can consider various criteria in a post-processing phase and examine the solutions without repeating the algorithm execution. Applying FastKnock to overproduce a number of biochemicals in E. coli genome-scale metabolic models, we found solutions that have not been reported by using state-of-the-art approaches.

## 1. Introduction

Metabolic engineering aims at the proper rewiring of cell metabolism to construct genetically engineered strains that can serve as robust cell factories for a variety of purposes, including the biosynthesis of target substances [1]. Extensive studies have been conducted in this field to develop methods for efficiently producing suitable natural compounds by using either native cells or heterologous hosts [2][3]. Systems metabolic engineering employs the concepts and capabilities of systems biology, synthetic biology, and evolutionary engineering at the systems level. It uses approaches from these disciplines and combines them with standard metabolic engineering techniques to facilitate the development of high-performance strains [4][5][6][7]. Metabolic systems biology plays a significant role in systems metabolic engineering because it incorporates a systems-level perspective on cellular metabolic functionalities [8][9][10][11]. Using metabolic systems biology, scholars can integrate omics data with results from genome-scale computational simulations to improve metabolic engineering techniques. These techniques can lead to the development of potentially productive and operationally optimized microbial strains [10][11][12][13].

The growth-coupled overproduction of (bio)chemicals is one of the most vital and practical objectives in systems metabolic engineering. Using this approach, synthesis of a desired compound can be guaranteed along with the reproduction of the engineered cell(s) [14][15]. Genome-scale metabolic network reconstructions (GENREs) [16] and their relevant mathematical representatives (genome-scale metabolic models (GEMs)) have been developed for numerous microorganisms (e.g., Escherichia coli [17][18][19][20], Pseudomonas putida [21][22], and Saccharomyces cerevisiae [23][24][25][26]). These tools are commonly used in computational systems biology for in silico production strain design. In particular, biased COnstraint-Based Reconstruction and Analysis (COBRA) computational techniques such as flux balance analysis (FBA) [27] and flux variability analysis (FVA) [28] are useful in analyzing GEMs [11][12][29] [30] (Supplement A). Using COBRA, one can take advantage of the synergistic effects of a variety of basic elements including genes, gene products and metabolites to evaluate cells’ potential and make model-driven discoveries. Accordingly, in silico studies based on systems-level analyses inspire researchers to examine intervention strategies, including gene or reaction insertions, knockouts, and up- or down-regulations [31][32]. For example, in several studies on gene and reaction knockouts, the candidates for the best combination of eliminations were identified [15][33][34][35][36].

There are two basic conventional approaches for designing metabolic intervention strategies: top-down (e.g., OptKnock [33], OptGene [37], MoMAKnock [34], CiED [38]) and bottom-up (e.g., FSEOF [39], CosMos [40]) procedures [41][42]. The top-down strategies are used to determine whether the potential interventions are advantageous and they iteratively search for the metabolic reaction network of interest until the optimal solutions are identified. The search space in the corresponding problems includes all combinations of a predefined number of reactions in a GEM. Due to the size of the developed and highly curated GEMs, this search space is extremely large and would explode with the cardinality of the combination. Thus, it would not be feasible to conduct an exhaustive exploration within a reasonable time frame.

Optimization techniques are commonly proposed to address this computational challenge. For example, OptKnock [33] is one of the most popular top-down frameworks. It uses bi-level optimization for *in silico* metabolic engineering. It aims to identify the appropriate sets of genes or reactions that, when knocked out, maximize the production rate of the desired biochemical coupled with biomass formation. To find an optimal solution for the growth-coupled production of the biochemical(s) of interest, OptReg [31] expands the capabilities of OptKnock by predicting appropriate up- or down-regulation of revealed crucial genes or reactions. RobustKnock [43] has been developed based on optimization techniques that guarantee the minimum production rate of the desired biochemical. Despite its novel approach, RobustKnock has not been widely used due to the difficulty of implementation.

The challenge in employing these optimization approaches is that the time required for finding an optimal solution grows exponentially with the cardinality of the combination. Worse, the solvers may fall into a deadlock situation and become trapped in an infinite loop. Several metaheuristic algorithms have been proposed to overcome this obstacle. These algorithms can pinpoint the suboptimal solutions within a reasonable time. For example, BAFBA [44] is a top-down metaheuristic method that deploys the bees algorithm [45] to find candidate gene knockouts and evaluate the results through FBA (Supplement A).

Bottom-up approaches discover appropriate intervention strategies by comparing two flux distributions. One of these distributions relates to the wild-type, which aims to maximize the cell’s growth rate. The other distribution relates to the functional state, which takes into account the goal of the desired biochemical overproduction. Examples include the flux distribution comparison analysis (FDCA) algorithm [46] and OptForce [32]. Using OptForce, all coordinated reaction modifications contributing to target overproduction are identified based on significant differences between the two flux patterns (initial and desired) in the introduced network, calculated using FVA. FVA finds the boundaries of the reaction fluxes that can satisfy the optimality of the solution under steady-state flux analysis (Supplement A).

In a nutshell, primitive top-down approaches use optimization methods to find an optimal solution at the cost of significant execution time. While top-down metaheuristic approaches require less computational resources, they are not guaranteed to find a globally optimal solution because the search space contains many local optima. On the other hand, bottom-up approaches can be used to find a set of potential solution candidates [14]. Despite various integrated computational and experimental studies, it is challenging to identify the most proper and operative alterations by only comparing the flux distributions of the wild-type to the ideally engineered states. Considering high order cardinalities and interventions [47] adds to the complexity of the problem.

State-of-the-art approaches have been developed to dramatically alleviate the computational challenges and significantly reduce the computational costs including (iteratively) pruning the search space [48][49] and sequentially enumerating the smallest minimal cut sets (MCSs) in order to provide several solutions [50]. For example, Fast-SL properly explores a metabolic network of interest to find the most appropriate synthetic lethal reaction sets. Fast-SL improves the performance of a brute-force search algorithm by iteratively reducing the size of the search space, which substantially shortens the execution time [49]. MCSEnumerator is another novel method that attempts to find many solutions using MCSs aimed at the identification of either synthetic lethal sets or optimal strain design targets [50].

Calculating the MCSs in GEMs is a complex and challenging computational problem [51]. The scalability of MCSEnumerator algorithms paves the way for both theoretical and practical studies considering high order simultaneous reaction interventions for strong growth-coupled product formation [52][53]. However, for *in silico* strain design, the MCSEnumerator approach require predefining of the acceptable thresholds for growth and target product yields and this contributes to different drawbacks such as neglection of some appropriate suboptimal solutions [54].

In this paper, we present FastKnock as a next-generation knockout strategy algorithm that provides the user with all possible solutions for multiple gene and reaction knockouts to overproduce a (bio)chemical of interest. Unlike the MCSEnumerator approach, FastKnock does not rely on any special parameter settings and additional assumptions (except for predefining the maximum number of simultaneous reaction knockouts). We developed a delicate search and prune algorithm to accomplish this goal at a greatly reduced computational time and cost. Our method combines (and benefits from) both basic approaches to tackle the problems described above. It incorporates reaction knockouts to couple the biosynthesis of both primary (e.g., succinate, lactate, ethanol, etc.) and secondary metabolites with cell reproduction. The secondary metabolites include native, e.g., dodecanoic acid, and heterologous biochemicals (e.g., polyketides such as erythromycin and terpenoids such as lycopene). It examines the GEM at the level of metabolic reactions while checking the corresponding genes to consider the gene dependency of the reactions.

The availability of all solutions allows us to systematically characterize and rank these strategies in accordance with some criteria including (a) substrate-specific productivity (SSP) [14][15][55][56], (b) the strength of growth coupling (SoGC), defined as the square of the product yield per unit substrate divided by the slope of the lower edge of the production curve [14][15][55][56], (c) strain dynamic performance, which depends on yield, productivity, and titer [57][58], and (d) other important indices reflecting environmental and operational considerations such as the feasibility of CO_2_ biofixation and minimal production of undesired or toxic byproducts. Some alternative criteria are discussed in [59]. Furthermore, it would be possible to evaluate the solutions and categorize them in the different major classes: potentially, weakly, directionally growth-coupled production (pGCP, wGCP, dGCP) and substrate-uptake coupled production (SUCP) raised in [60].

This article is organized as follows: Section 2 introduces the FastKnock algorithm, which we designed to effectively search the metabolic network to find all reaction knockout strategies that result in the overproduction of the desired biochemical(s). Section 3 presents the results of *in silico* experiments employing highly curated GEMs of *E. coli*. Last, Section 4 presents our conclusions.

## 2. The proposed method

We developed the *FastKnock* algorithm, which is a general framework that can be used to increase the production rate of the desired metabolite in a cell simultaneously with growth. The desired metabolite can be of a primary or secondary type and can be native or heterologous. Specifically, the algorithm can be applied on heterologous metabolites through the inclusion of the associated pathways into the GEM set.

In other words, FastKnock identifies reactions to be deleted from the network while ensuring that the flux of biomass formation reaction remains above a specific cut-off (i.e., 1% of *gr*_*WT*_, Supplement D) and the production of the desired substance(s) increases as much as possible [61]. For practical applications, FastKnock can be used to find the subsets of network reactions that can be removed in order to significantly increase the production of the desired biochemical. Specifically, FastKnock identifies the strains in which the production rate of the desired biochemical is more than a predefined threshold in the base model (i.e., the model without any interventions). We call this threshold *Th*_*chemical*_, which we define as 5% of the maximum theoretical yield (i.e., the optimal production rate of the desired biochemical when it is considered the objective of the cell) in the base model. FastKnock, like other common approaches, uses preprocessing to reduce the size of the metabolic model reactions and the search space. In the preprocessing phase (Supplement C), the set of the removable reactions (denoted by *Removable*) is identified and structurally excluded from the metabolic network to produce a reduced model denoted as *Reduced_model*. The set of reactions of the *Reduced_model* is called *RXNS*.

The search space of the exhaustive search includes all sets of reactions of the *Reduced_model* with a particular size. This search space grows exponentially as the size of the set increases. Therefore, examining all sets using an exhaustive search is very time-consuming and would be infeasible. To tackle this problem, our proposed algorithm uses the information that is available only during the search procedure to dynamically narrow the search space (i.e., the search space is iteratively pruned and some reactions are temporarily excluded). This reduced search space is used to find the knockout strategies; therefore, we call it the *target space*.

For practical applications, one important feature of FastKnock is that it can optionally consider genes as the basis of reaction deletions. This is a realistic assumption because knocking out genes to remove a specific reaction often leads to removing a predetermined set of reactions that are simultaneously knocked out. In this work, we label a set of reactions as *co-knocked out* if they are removed due to the elimination of a single gene. Supplement E explains a modification of the algorithm based on knocking out genes rather than reactions.

### 2.1 FastKnock algorithm

Our proposed method aims to find all solutions to a strain optimization problem to achieve the growth-coupled overproduction of a metabolite (i.e., biochemical) of interest. Each solution is a set of *k* reactions (i.e., a knockout strategy) such that the elimination of these reactions creates a new engineered strain in which the overproduction of the biochemical of interest is coupled with cell growth.

Testing whether a set of reactions is a proper solution is equivalent to solving an optimization problem in which the objective function is the growth of the cell and reactions elimination corresponds to adding constraints to the optimization problem. By solving this optimization problem, we obtain the flux of all the reactions including the production rate of a desired biochemical. An appropriate solution (i.e., a knockout strategy) should satisfy the objective function along with providing a suitable production rate for the desired biochemical product.

To find all subsets of reactions of size ≤ k, we consider a tree-based representation of all the combinations of reactions with a maximum size of k, which is outlined below. All sets of k reactions are placed in nodes of the tree in depth k (i.e., at the level k). The root node at level zero corresponds to removing no reaction (i.e., wild-type microorganism). The FastKnock procedure starts with investigating the elimination of a single arbitrary reaction r1 at level one. Whether knocking out r1 is a solution or not, we continue investigating simultaneous elimination of r1 and another reaction at level two. At each level, we consider *only* the reactions that have non-zero flux according to the optimization problem solved in the parent node in the upper level. The procedure of adding reactions with non-zero flux to the set of knockout reactions continues at lower levels of the tree until one of the two stopping conditions is met: a) we reach a leaf at level k (the predefined number of knockouts) or b) we reach a node that is guaranteed to have no solution in its subtree.

To check condition b in each node at level l<k, we determine whether the subtree may not include a solution by investigating the optimization problem. Specifically, if the optimization problem already has an infeasible region at a node, adding more constraints in the subtree of the node would not lead to a proper solution (Supplement F).

The merit of the procedure is the technique of bounding the search by a) excluding the reactions with zero flux at each node and b) checking the feasibility of reaching a solution *before* expanding the subtree of each node. This way, we dynamically and effectively prune the search space.

**Figure 1:**
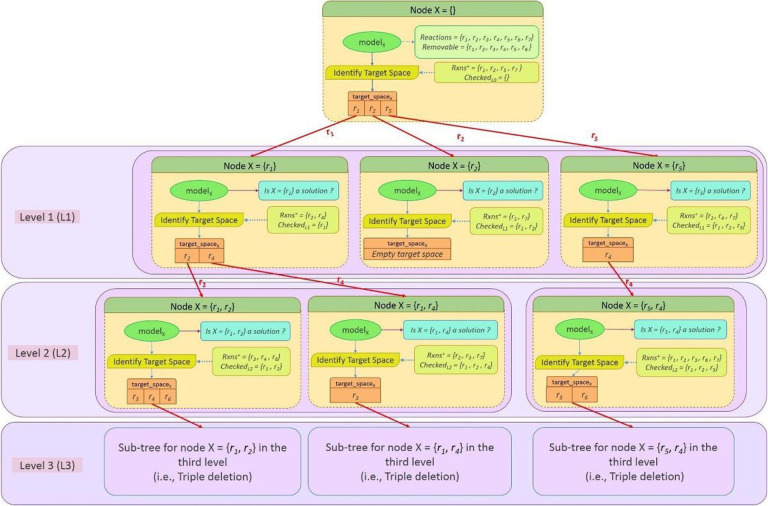
The traversal tree. All possible solutions are identified through a depth-first traversal of the tree. First, the identifyTargetSpace function is applied in the root node to the reduced wild-type network to determine the target space. Each reaction in this set is individually selected and removed from the network in Level 1. For each deleted reaction (or equally node) in Level 1, the identifyTargetSpace function is recalled to obtain the target space for the next level. For simplicity, we show only two levels of the traversal of the tree, which is enough to identify all single and double deletions.

Figure 1 illustrates the overall procedure using a depth-first traversal tree. The root node corresponds to the base model in which no reaction is deleted. Algorithm 1 represents the definition of a node in the tree, as well as, the main procedure of the FastKnock algorithm. Each instance of the *Node* contains the model, the set of the removed reactions, the search space, and the target space for the next level (Figure 1). Specifically, at each node *X* of the tree at level *L*, we investigate a set of L reactions (*deleted_rxns*) to determine (a) whether *X* is a solution and (b) the new target space, which is the set of all reactions that could potentially be added to *deleted_rxns* for investigation at the next level.

Determining the target space at each node is critical, and it allows us to avoid the combinatorial explosion of the tree that would inevitably result from an exhaustive search. In particular, while we investigate drastically fewer subsets of reactions at the children nodes in Level *L*+1, our analysis guarantees that FastKnock will find every candidate solution (Supplement F).

In Algorithm 1, the traversal of the tree shown in Figure 1 is represented by a set of queues: *queue*_*1*_ to *queue*_*target_level*_. Each queue contains a set of nodes. At each moment during the execution of the algorithm, queue *l* contains all children of a certain node at level *l*-1 being investigated. In this way, the subtrees are gradually constructed and removed (pruned).

The main algorithm consists of three functions: *identifyTargetSpace, constructSubTree*, and *traverseTree*. For each node, we compute a target space and a flux distribution using the *identifyTargetSpace* function. This function temporarily narrows the search space for the whole subtree of the node. The subtree of a node is constructed using the *constructSubTree* function. The *traverseTree* function recursively navigates the tree, based on a depth-first traversal.

We elaborate these functions in the following subsections. First, we determine the target space and then describe the search procedure (i.e., how the traversal tree is partially constructed and traversed). In our implementation, we improved the quality of the obtained solutions by guaranteeing the minimal chemical production rate (Supplement I), and increased the speed of the algorithm using parallel processing (Supplement G).

#### 2.1.1 Identifying the target space

At steady state, a specific flux range for each reaction r is obtained (*minFlux*_*r*_ ≤ *f*_*r*_ ≤ *maxFlux*_*r*_), which leads to the optimal cellular objective (e.g., maximizing the biomass formation flux). Knocking out a reaction *r* is implemented by setting the allowable flux range [62] of the reaction to zero (i.e., lb_r_ = ub_r_ = 0 in the optimization problem of Equations a.1 and a.5 in Supplement A). Note that when a reaction is reversible (i.e., the obtained flux range of a reaction includes zero minFlux_r_ ≤ 0 ≤ maxFlux_r_), knocking out that reaction alone has no effect on the optimal objective value of the network (Supplement F).

Here, the main idea is to prune the target space by considering only the set of reactions with nonzero flux values. This approach significantly reduces the size of the target space and thus reduces the execution time of the algorithm.

We denote reactions that lack a zero value in their obtained flux range as *Rxns*^+^ in each node of the tree:

$${\text{Rxns}}^{+}=\{r\in \text{Rxns}|{\text{minFlux}}_{r}>0\;\text{or}\;{\text{maxFlux}}_{r}<0\}.$$

The target space of each node, which is the set of reactions that could be appropriate for deletion, is obtained using the *identifyTargetSpace* function (Algorithm 2). The search operation at each node is limited to *Rxns*^+^ ∩ *Removable*, as shown in Line 6 of Algorithm 2.

It is worth mentioning that by any manipulation of the model, the fluxes of other reactions may change. Therefore, the functional states (i.e., flux distribution) should be analyzed repeatedly after each modification (i.e., after each reaction knock-out) using FBA to identify the reactions that carry non-zero flux in the network (*model*_*X*_) (Lines 4–5). The *flux_dist* variable of the node is updated at Line 4. The intersection of these reactions and the *Removable* set construct the target space of node *X* in Line 6.

#### 2.1.2 The search procedure

Here, we introduce a depth-first search procedure based on the traversal tree of Fig 1. Each node of the tree has its own subtree, which is traversed before traversing its sibling nodes. This *depth-first search procedure* is implemented using the *traverseTree* function of Algorithm 4.

In each call, the *traverseTree* function visits a certain node *X* (i.e., the first node of the *queue*_*level*_) and, if needed, calls the *constructSubTree* function to create the corresponding subtree of the node (Algorithm 3). The *constructSubTree* function creates the children nodes of X, which is a set of nodes that are placed n *level* = X.*level* + 1. For each child, *deleted_*rxn*s* is initialized by adding one of the reactions in *X.traget_space* to the *X.deleted_rxns*.

It is clear that the order of the knocked-out reactions is not important. In FastKnock, repetitive permutations of the reactions are ignored using a *checked*_*level*_ queue for each level of the tree. Generally, *N* levels are considered for simultaneously knocking out *N* reactions from the cell. Precisely, the reaction selected for the *i*^*th*^ level is not allowed in the *(i+1)*^*th*^ to *N*^*th*^ levels. To generate all combinations of these reactions, the *checked*_*L*_ queue is used at level *L*. At level *L*, by deleting a reaction *r* from the target space, *r* is added to the *checked*_*L*_. This excludes the reaction from the target space of the subsequent levels.

## 3. Results

We implemented the FastKnock algorithm using Python language programming (Version 2.7) and the COBRApy library (Version 0.15.4) [63]. Our source code is publicly available at https://github.com/leilahsn/FastKnock and also as supplementary material. We evaluated the performance of FastKnock using various examples, and we compared these results to an alternative approach.

### 3.1 FastKnock results for *E. coli* models

To evaluate FastKnock’s performance, we selected three highly curated GEMs for *E. coli* (i.e., iJR904 [17], iAF1260 [18], and iJO1366 [19]) for our experiments to overproduce some well-known metabolites, including succinate, lactate, 2-oxoglutarate, and lycopene as the both primary and secondary biological products.

We tested the production of primary metabolites focusing on two cultivation conditions: The first condition is *CM1*: *i*M9 medium supplemented with glucose (a maximum allowable glucose uptake rate of 10 mmol.gDW^−1^h^−1^) under aerobic conditions (a maximum allowable oxygen uptake rate of 15 mmol.gDW^−1^h^−1^). The second condition is *CM2*: *i*M9 medium supplemented with glucose (a maximum allowable glucose uptake rate of 10 mmol.gDW^−1^h^−1^) under anaerobic conditions (an oxygen uptake rate of 0 mmol.gDW^−1^.h^−1^).

Many of the models’ reactions are not active in the minimal *i*M9 medium. In a complex and rich environment, due to the activation of more inputs to the cell, more pathways and consequently more reactions are active in the network. Hence, in order to further evaluate the exhaustive enumeration performance of the FastKnock algorithm in a rich cultivation condition, we conducted additional *in silico* experiments considering Luri-Bertani (LB) medium. The *i*LB medium constraints were intended based on [64], [65]. We deployed the two highly- curated *E. coli* GEMs (i.e., iJR904 and iML1515 [20]) for the experiments. The input settings (i.e., exchange fluxes) to define the mediums for the models used in the current study are listed in the exchanges.xls file.

The secondary metabolite, lycopene, is produced in the cell only under aerobic conditions. We considered two strains for lycopene production. For the first strain (*Strain1*), the lycopene biosynthesis pathway (i.e., the methylerythritol phosphate (MEP) pathway [66]) is added to the wild-type *E. coli* model [39][67] [68]. For the second strain (*Strain2*), some other modifications are applied based on [69]. This provides an intracellular pool of pyruvate as the important precursor of lycopene production [70]. Tables 1 and 2 in Supplement J.I show the maximum theoretical yield for the biosynthesis of the metabolites (i.e, maximum of *V*_*chemical*_) and our threshold for their production (*Th*_*chemical*_ = 0.05 *V*_*chemical*_).

The result of the preprocessing phase is shown in Table 2 of Supplement J.I, which demonstrates the number of reactions that are excluded from the search space before the main exploration procedure is applied and before the removable reactions are obtained. The size of the search space is drastically reduced to 20% of all the reactions. In the *Reduced_model*, the blocked reactions and dead ends are removed [62]. Also, as described in Section 2, after the preprocessing phase, the search space is reduced iteratively and temporally during the search procedure of the FastKnock algorithm. This significantly reduces the number of linear programming problems (LPs) that must be solved. Specifically, compared to an exhaustive search, the reduction rates are 78%–85% for single knockouts, 95%–97% for double knockouts, 99.0–99.5% for triple knockouts, and above 99.8% for quadruple and quintuple knockouts (Table 1). The number of LPs is equal to the number of nodes in the traversal tree shown in Figure 1, and it is independent of the target metabolite to be produced.

In comparison, in the exhaustive search the algorithm must check all the combinations of the reactions in the search space. For instance, iJR904 in CM2 has 208 reactions in its search space. For finding double-knockout results in the exhaustive search, the algorithm must check all the double combinations of the elements in the search space (c(208, 2) = 21,528). Due to its time complexity, the exhaustive approach is not feasible for high-order reaction knockouts; thus, we compared FastKnock to a simple exhaustive search method for single, double, or triple knockouts. Our experiments showed that a significant reduction in the number of LPs is critical because it allows us to investigate and find all possible solutions.

Table 2 presents the total number of solutions obtained using the FastKnock algorithm. The results are reported in two cases: the maximum production rate (*Rate*_*max*_) and the guaranteed production rate (*Rate*_*grnt*_) as discussed in Supplement I.

We also compared our solutions to the results of the exhaustive search for single, double, and triple deletions for succinate production in iJR904 to verify the completeness of the FastKnock algorithm. Both approaches found two solutions for a single deletion. The exhaustive search for a double deletion found 398 solutions, of which only 58 solutions were true double deletions. The rest of the solutions were not acceptable because either (a) the combination of each single deletion solution and a zero-flux reaction was inappropriately considered as a double-deletion solution or (b) the elimination of a reaction in the co-knocked-out sets led to the removal of all the reactions in the set, while in the exhaustive search, the removal of each reaction in the set is counted as a separate solution. For triple deletions, the exhaustive search found 39,407 solutions, of which 887 were unique and acceptable. FastKnock found all the 887 solutions.

**Figure 2:**
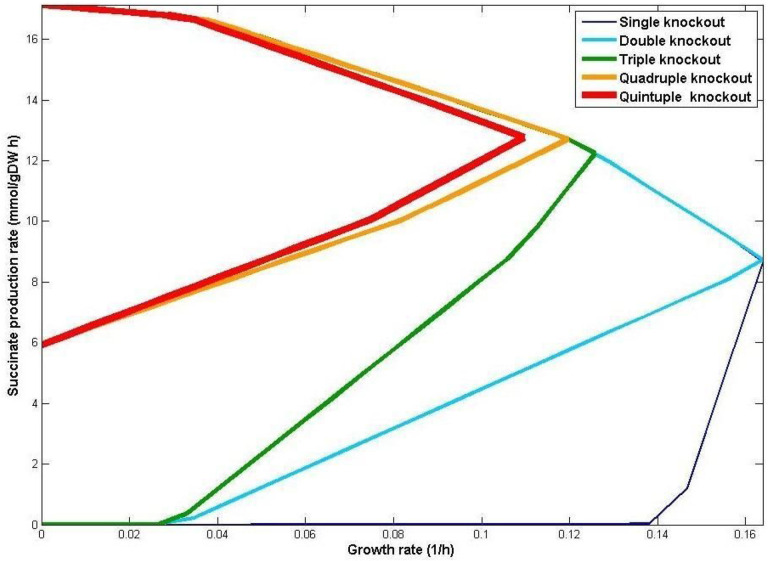
Production envelopes for the best solutions presented in Table 3 regarding succinate production from single to quintuple reaction deletions in iJR904. Knocking out more genes improves growth coupling. In particular, with quadruple and quintuple knockouts, significant production is guaranteed for any growth rate.

Table 3 presents the best solutions in iJR904 GEM as *Rate*_*grnt*_ mode. Supplement J.II shows the results for the iAF1260 and iJO1366 GEMs as well as the maximized solutions. As an example, we found that the best result for succinate overproduction is obtained by deleting one reaction, ADHEr, which is knocked out by the deletion of the gene b1241. Consequently, the deletion of the b1241 gene also causes the deletion of the LCADi_copy2 reaction. In this situation, the growth rate is 0.16 (1/h) as shown in the biomass formation rate column. After the deletion of ADHEr, the succinate production can vary between 5.11 and 9.50 mmol.gDW^−1^h^−1^, which is more than the 0.85 mmol.gDW^−1^h^−1^ threshold; hence, an acceptable amount of succinate production is guaranteed. Moreover, Table 3 presents the production envelopes calculated for succinate production (Figure 2).

For practical applications, various evaluation indices, including product yield, SSP, and SoGC [55], and other important indices reflecting environmental and operational considerations, can be used to choose the most appropriate cases from the solutions found by FastKnock (Table 4 and Table 5). In particular, the feasibility of CO_2_ biofixation and minimal production of undesired or toxic byproducts are also significant indexes for systems metabolic engineering purposes. For instance, an engineered strain that can simultaneously fix CO_2_ and produce a suitable biochemical might be preferred regarding environmental considerations. When all solutions are available, the analysis and identification of such appropriate cases is easily possible.

We analyzed FastKnock solutions in order to find the most appropriate solutions based on three criteria, yield, SSP, and SoGC (Table 5). Additionally, the feasibility of CO_2_ biofixation is also examined and the relevant results are summarized, where a negative CO_2_ exchange flux represents a desirable CO_2_ uptake rate. We compared these best solutions obtained by FastKnock with the associated OptKnock results as well as experimental data available in the literature [71][72][73]. Note that Optknock aims at, and terminates on, finding a single solution. Therefore, comparing it vs. FastKnock in terms of computational costs is not meaningful.

We found that a solution with the best production rate or an optimal solution of the optimization algorithms such as OptKnock does not necessarily bring the best SoGC and the other desired indexes. However, by identifying all the possible solutions for the problem, FastKnock allows a comprehensive analysis. For example, knocking out ADHEr, ATPS4r, and LDH_D is expected to lead to the best biomass formation rate (0.16 h^−1^) and the highest SoGC (3.01 h^−1^), which is twice the best SoGC provided by OptKnock solutions while the other indices corresponding to this knockout are comparable with the best numbers shown in the table (i.e., a production rate of 8.90 vs. 12.24 mmol.gDW^−1^.h^−1^, a yield of 0.89 vs. 1.22, an SSP 1.42 vs. 1.46 h^−1^, and a CO_2_ uptake rate of −8.76 vs. −9.36 mmol.gDW^−1^.h^−1^). A relatively high value of SoGC can also be desirable from a dynamic perspective because it indicates that even under non-optimal conditions, the biosynthesis of the target biochemical is coupled with the growth of the production strain. This situation is usually encountered in batch and fed-batch cultivations in the logarithmic phase of growth.

A more striking example is the comparison between the PTAr, PYK, ATPS4r, and SUCD1i quadruple knockout identified by OptKnock with the two solutions with the best production rate (ADHEr, LDH_D, PFL, and THD2) and the best SoGC (ADHEr, LDH_D, HEX1, and THD2) identified by FastKnock. While the biomass formation rate of the FastKnock solutions (0.11, 0.13 h^−1^, respectively) are comparable with the OptKnock solution (0.16 h^−1^), the yield and SSP is an order of magnitude higher for FastKnock solutions. A serious issue with this OptKnock solution is the very low SoGC (0.01 h^−1^), which indicates that the production rate would be hardly coupled with growth. In comparison, the predicted SoGC for FastKnock solutions are 2.85 and 3.09 h^−1^, respectively. Another disadvantage of OptKnock solution is a relatively high CO_2_ production rate of 9.03 mmol.gDW^−1^.h^−1^ while in the FastKnock solutions the CO_2_ uptake rates are −6.12 and −8.77 mmol.gDW^−1^.h^−1^, respectively.

Among the quintuple knockouts, the predicted SSP and SoGC for one of the FastKnock solutions (ADHEr, LDH_D, GLUDy, PFL, and THD2) are almost twice those of the OptKnock solution (ADHEr, LDH_D, PTAr, PYK, and GLCpts) while the other indices are comparable.

### 3.2 Comparing FastKnock to MCSEnumerator (case study: ethanol overproduction in *E. coli AF1260*)

As mentioned previously, MCSEnumerator is a novel method for metabolic engineering based on the identification of minimal cut sets [50]. This approach applies a filtering step to reduce the computation time, which allows the user to find thousands (but not all) of the most efficient knockout strategies in genome-scale metabolic models. MCSEnumerator can be used to find a large number of metabolic engineering interventions, but it has various drawbacks. In this section, we compare MCSEnumerator with FastKnock. To aid in this comparison, we consider the case study of ethanol production in *E. coli* i*AF1260* GEM with an 18.5 mmol.gDW^−1^h^−1^ glucose uptake rate under anaerobic conditions (*i*M9 medium) as presented in the MCSEnumerator publication.

**Figure 3:**
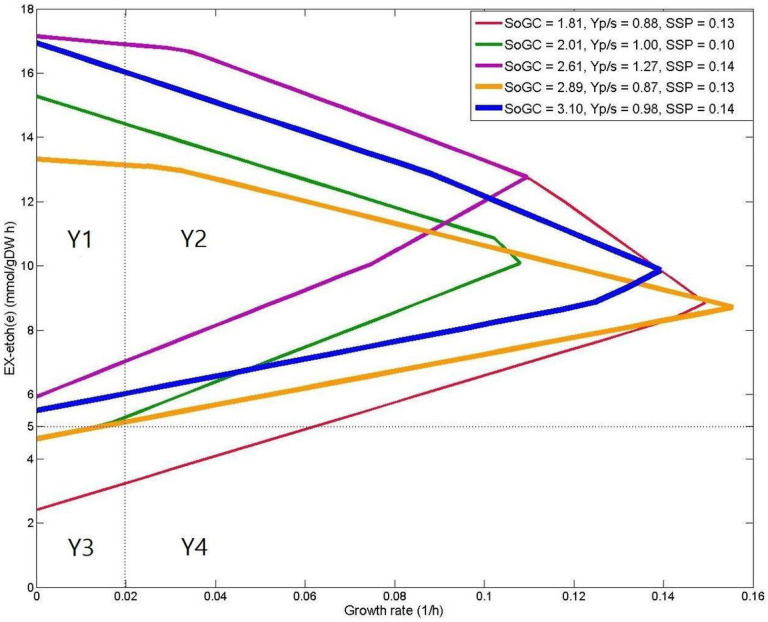
Five exemplar production envelopes for strategies identified by FastKnock for ethanol production in iAF1260, which is partitioned into four regions based on the growth rate (x axis) and the production flux (y axis) as in [15]. The horizontal dashed line indicates the threshold for production rate considered in [15], and the vertical dashed line indicates the growth rate threshold. SoGC, product yield (Yp/s) and SSP of the quadruple knockout strategies are shown in the top right legend. Unlike FastKnock, MCSEnumerator finds none of these strategies except the one shown in blue.

We should discuss the effect of the MCSEnumerator thresholds on its solution set. It would not be feasible to apply MCSEnumerator using thresholds that are relaxed enough to find all the solutions (Supplement H). We illustrate this with an example in Figure 3. The blue production envelope, which has the best SoGC value, is associated with a solution found by both MCSEnumerator and FastKnock. The associated solutions (with the red and green diagrams), which are the worst cases among the shown envelopes, were not found by MCSEnumerator because of the production threshold considered. This illustrates the efficiency of the primary filtration of the MCSEnumerator method. The starting point might not be the best factor for filtering appropriate solutions. For example, the minimum production rate based on the orange envelope is similar to the green envelope in Region Y3, which is below the threshold considered for ethanol production flux. Nevertheless, the orange envelope may still be associated with a proper solution due to its relatively high SoGC, but it was not found by MCSEnumerator.

Furthermore, the predetermined thresholds may lead to the fact that some of the solutions obtained by MCSEnumerator are not necessarily and truly minimal. It means that an appropriate solution of cardinality (n) may exist and not be found while it appears in some higher-order solutions (>n) which contain irrelevant additional reactions.

While the MCSEnumerator algorithm and its modified editions may have shorter execution times, the number of solutions they can provide with certain settings is only a very small percentage of the total potential solutions. Therefore, comparing the MCSEnumerator and FastKnock algorithms based solely on execution time is not rational since these algorithms neither produce the same output nor have the same objective.

## 4. Discussion

Overproduction of biochemicals of interest coupled with significant growth rates might be optimistic and may not always be easily achievable due to e.g., competing pathways in a metabolic network [43]. This can lead to weak coupling especially under suboptimal growth conditions. Alternatively, strong coupling requires that production must occur even without growth [14]. Specifically, product synthesis rate is said to be strongly coupled with biomass formation if the product yields of all steady-state flux vectors are equal to or larger than a predefined product yield threshold [15]. Accordingly, SoGC is defined as the square of the product yield per unit substrate divided by the slope of the lower edge of the production curve [55] (see Figure 2).

SoGC is a non-linear objective function and thus OptKnock and most of the in silico strain design methods cannot be used to find knockouts with optimal SoGC. OptGene [37] is a heuristic approach that can be used to identify a single knockout strategy with optimal SoGC [55]. However, knocking out the single identified solution by OptGene may not be practically feasible e.g, due to the genes’ loci. Therefore, identification of all knockout strategies by FastKnock is desired and provides the expert experimentalists with the opportunity to choose from a short list of knockout strategies that are filtered for a relatively high SoGC, SSP, yield, etc. This shortlist can be investigated for advantageous solutions in terms of environmental considerations such as CO_2_ biofixation [71][72], minimal production of undesired or toxic byproducts, practicality of knocking or silencing genes, etc (Table 5) [6][55][73][74][75].

We proposed an efficient next-generation algorithm, FastKnock, which identifies all proper reaction or gene knockout strategies for the overproduction of a desired biochemical. We reached this goal by significantly pruning the search space without omitting any solutions. For example, in our experiments, FastKnock was required to explore only 1% of the search space in the pruned model when identifying all triple-knockout strategies. The rate of this reduction increases as more reactions are knocked out (e.g., about 0.1% for quadruple-knockout strategies and about 0.01% for quintuple-knockout strategies) (Table 3). This drastic reduction of the search space enables our novel FastKnock method to find the set of all possible solutions in a feasible time duration.

Finding the best and most suitable trade-off between cellular growth and the production of the desired biochemical is one of the key benefits of FastKnock results. Moreover, determining all possible solutions allows for the selection of the most appropriate strategy based on any desired evaluation index, including product yield, SSP, and SoGC (Table 4 and Table 5). This is an important and useful feature of our search strategy, especially for practical applications [59].

We compared FastKnock to MCSEnumerator [50], which has been shown to find more efficient solutions than the MCS methods [76][77][78]. We found that the solutions identified by MCSEnumerator may not be minimal. Also, due to initial filtering, MCSEnumerator misses solutions that may be practically more appropriate than the best solutions it finds. In comparison, FastKnock identifies all minimal solutions, which can be mined later based on any desired criteria.

**Figure 4:**
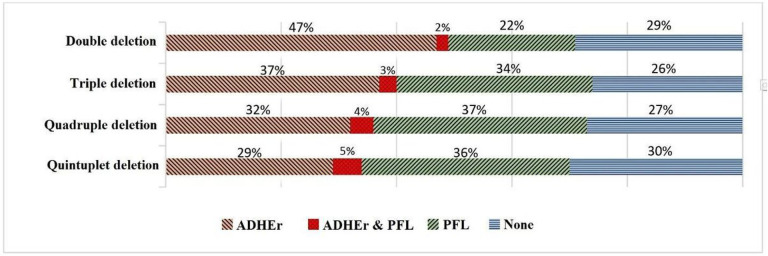
The rate of presence of the ADHEr and PFL reactions in all possible solutions counted in Table 4 for succinate production.

When all solutions are available, one interesting analysis that can be conducted is to identify the reactions or genes that are common among a relatively large number of solutions. For instance, in the case of iJR904, to produce succinate in *i*M9 under anaerobic conditions (*CM2*), about 70% of solutions include at least one of *ADHEr* or *PFL* reactions (Figure 4). Moreover, when three or more reactions are to be deleted, the best results in terms of the succinate production rate include both *ADHEr* and *PFL* (Table 4). Collectively, this analysis suggests that *ADHEr* and *PFL* reactions support pathways that compete with succinate production, and these pathways are blocked when *ADHEr* and *PFL* are eliminated [79][80]. Based on this analysis, we suggest using a heuristic for higher-level knockout combinations in which one or more reactions (e.g., *ADHEr* or *PFL*) are removed in searches for six or more knockouts. In this way, one would need to search for fewer reactions to knockout. We believe this heuristic would reduce the search space by an order of magnitude at the expense of losing not more than half of the solutions.

## 5. Conclusion

While *in silico* results do not necessarily lead to *in vivo* overproduction, obtaining all possible knockout strategies is critical for determining the best practical and most efficient strategy. The FastKnock algorithm is a general framework that can be used to overproduce any metabolite. It is not limited by factors such as complexity of the cultivation conditions or large size of the metabolic network of the desired strain. FastKnock identifies strategies with a production rate higher than the desired threshold determined by the user.

## Figures and Tables

**Table 1:** Number of linear programming problems (LPs) solved by the FastKnock algorithm compared to an exhaustive search of the preprocessed search space.

			Single	Double	Triple	Quadruple	Quintuple
**CM2**	**iJR904**	Exhaustive search	208	21,528	1,478,256	75,760,620	3,091,033,296
		FastKnock	41	820	11,613	125,815	1,178,030
		**% Reduction**	**80.29**	**96.20**	**99.22**	**99.84**	**99.97**
	**iAF1260**	Exhaustive search	315	49,455	5,159,805	402,464,790	25,033,309,938
		FastKnock	57	1,506	25,985	348,966	4,058,061
		**% Reduction**	**81.91**	**96.96**	**99.50**	**99.92**	**99.99**
	**iJO1366**	Exhaustive search	385	73,920	9,437,120	901,244,960	68,674,865,952
		FastKnock	58	2,038	43,565	732,315	10,822,208
		**% Reduction**	**84.93**	**97.24**	**99.53**	**99.91**	**99.98**
**Strain2**	**iJR904**	Exhaustive search	237	27,966	2,190,670	128,154,195	5,971,985,487
		FastKnock	50	1,159	17,330	207,683	2,230,192
		**% Reduction**	**78.90**	**95.85**	**99.20**	**99.83**	**99.96**
	**iAF1260**	Exhaustive search	347	60,031	6,903,565	593,706,590	40,728,272,074
		FastKnock	62	1,832	35,913	537,703	6,930,724
		**% Reduction**	**82.13**	**96.94**	**99.47**	**99.90**	**99.98**
	**iJO1366**	Exhaustive search	410	83,845	11,402,920	1,160,247,110	94,212,065,332
		FastKnock	69	2,354	53,222	932,688	14,414,728
		**% Reduction**	**83.17**	**97.19**	**99.53**	**99.91**	**99.98**

**Table 2:** Number of solutions in iJR904

Order of reaction knockout	CM2						Strain2	
	Succinate		2-Oxoglutarate		D-lactate		Lycopene	
	*Rate*_*max*_	*Rate*_*grnt*_	*Rate*_*max*_	*Rate*_*grnt*_	*Rate*_*max*_	*Rate*_*grnt*_	*Rate*_*max*_	*Rate*_*grnt*_
**Single**	2	1	0	0	0	0	0	0
**Double**	58	27	0	0	10	7	0	0
**Triple**	887	416	0	0	308	228	0	0
**Quadruple**	10090	4794	0	0	4941	3790	4	0
**Quintuple**	98300	48693	29	0	58481	13639	154	4

**Table 3:** Guaranteed production rates for succinate (Rate_grnt_) in iJR904 in CM2 medium for succinate production

Number of knockouts	Deleted reaction	Biomass formation rate (h^−1^)	Production rate (mmol.gDW^−1^.h^−1^)		SoGC (h^−1^)	Deleted genes	Co-knockout reactions
			min	max			
**Single**	ADHEr	0.16	5.11	9.50	1.41	b1241	LCADi_copy2
**Double**	ADHEr, LDH_D	0.15	8.08	9.51	1.43	b1241, b2133, b1380	LCADi_copy2
**Triple**	ADHEr, LDH_D, PFL	0.12	11.08	12.73	1.53	b1241, b2133, b1380, b3114, b0902, b3951	LCADi_copy, OBTFL
**Quadruple**	ADHEr, LDH_D, PFL, THD2	0.11	12.29	13.01	2.58	b1241, b2133, b1380, b3114, b0902, b3951, b1602	LCADi_copy, OBTFL
**Quintuple**	ADHEr, LDH_D, GLUDy, PFL, THD2	0.10	12.34	13.06	2.61	b1241, b2133, b1380, b1761, b3114, b0902, b3951, b1602	LCADi_copy, OBTFL
*Table 3–1: Maximized production rates for succinate (Rate*_*max*_ *) in iJR904 in rich medium (LB) for succinate production*							
Number of knockouts	Deleted reaction	Biomass formation rate (1/h)	Production rate (mmol*gDW^−1^*hr^−1^)			Deleted genes	Co-Knockout reactions
**Single**	ADHEr	1.35	20.10			b1241	LCADi_copy2
**Double**	F6PA, PFK	1.28	33.69			b0825, b3946, b3916, b1723	-
**Triple**	ACKr, GLCpts, PYK	0.56	54.88			b2296, b3115, b1849, b1819, b2415, b2416, b1621, b1101, b2417, b1817, b1818, b1854, b1676	DHAPT, GART, PPAKr
**Quadruple**	ACKr, ARGDC, GLCpts, PYK	0.56	64.72			b2296, b3115, b1849, b2938, b4117, b1819, b2415, b2416, b1621, b1101, b2417, b1817, b1818, b1854, b1676	GART, PPAKr, DHAPTs
*Table 3–2: Maximized production rates for succinate (Rate*_*max*_ *) in iML1515 in CM2 medium for succinate production*							
Number of knockouts	Deleted reaction	Biomass formation rate (1/h)	Production rate (mmol*gDW^−1^*hr^−1^)			Deleted genes	Co-Knockout reactions
**Single**	ATPS4rpp	0.25	12.73			b3735, b3737, b3738, b3732, b3733, b3736, b3734, b3731, b3739	-
**Double**	ATPS4rpp, PGL	0.24	16.54			b3735, b3737, b3738, b3732, b3733, b3736, b3734, b3731, b3739, b0767	-
**Triple**	PGI, ATPS4rpp, G6PDH2r	0.17	23.16			b4025, b3734, b3733, b3736, b3732, b3737, b3731, b3738, b3739, b3735, b1852	-
**Quadruple**	PFL, ACALD, THD2pp, THD2pp	0.19	23.49			b0351, b1241, b0903, b3951, b2579, b3952, b3114, b0902, b1602, b2913	OBTFL, ‘ALCD2x’, ‘ALCD19’
*Table 3–3: Maximized production rates for succinate (Ratemax) in iML1515 in rich medium (LB) for succinate production*							
Number of knockouts	Deleted reaction	Biomass formation rate (1/h)	Production rate (mmol*gDW^−1^*hr^−1^)			Deleted genes	Co-Knockout reactions
**Single**	ARGDC	1.08	19.72			b4117	-
**Double**	ARGDC, FADRx	1.05	22.09			b4117, b3844	FADRx, FE3Ri, FLVRx
**Triple**	NDPK5, ASPTA, ARGDC	1.03	28.14			b0474, b2518, b0928, b4054, b4117	ADK1, NDPK2, ADNK1, NDPK3, NDPK6, DADK, ADK4, NDPK1, ADK3, NDPK4, NDPK7, NDPK8, TYRTA, PHETA1, LEUTAi
**Quadruple**	NDPK5, PFL, LDH_D, ACALD	0.75	40.97			b0474, b2518, b2579, b3952, b0902, b3951, b0903, b3114, b1380, b0351, b1241	ADK1, NDPK2, ADNK1, NDPK3, NDPK6, DADK, ADK4, NDPK1, ADK3, NDPK4, NDPK7, NDPK8, OBTFL, ALCD2x, ALCD19

**Table 4:** The best solutions based on the desired evaluation indexes for succinate production under anaerobic condition in iJR904

	Evaluation index								
	SSP				linearMOMA				SoGC
Number of knocked out reactions	Best knockout strategy	FBA biomass (h^−1^)	FBA succinate rate (mmol.gDW^−1^.h^−1^)	Biomass * succinate rate (mmol.gDW^−1^.h^−1^)	Best knockout strategy	MOMA biomass (h^−1^)	MOMA Succinate rate (mmol.gDW^−1^.h^−1^)	Biomass * succinate rate (mmol.gDW^−1^.h^−1^)	Best knocko strateg
1	ADHEr	0.16	0.83	0.14	ADHEr	0.03	2.38	0.08	ADHEr
2	ADHEr, LDH_D	0.16	8.73	1.43	ADHEr, ATPS4r	0.12	8.32	1.01	ADHEr LDH_D
3	ADHEr, LDH_D, PFL	0.12	12.24	1.53	ADHEr, ATPS4r, RPE	0.13	8.60	1.19	ADHEr ATPS4 LDH_D
4	ADHEr, LDH_D, PFL, URIK2	0.12	12.24	1.53	ADHEr, ATPS4r, LDH_D, RPE	0.13	8.71	1.20	ADHEr LDH_D HEX1, THD2
5	ADHEr, P, PFL, SUCOAS, RNDR3	0.12	12.25	1.54	ADHEr, ATPS4r, GLYK, F6PA, RPE	0.14	8.63	1.23	ADHEr LDH_D HEX1, THD2, DRPA

**Table 5:** Comparison of FastKnock, OptKnock and experimental results from the literature for succinate production. The iJR904 model is used in the in-silico experimentations.

Knockout	Knockout strategy	method	Biomass formation rate (h^−1^)	Production rate (mmol.gDW^−1^.h^−1^)	yield	SSP (h^−1^)	SoGC (h^−1^)	CO_2_ uptake / production rate (mmol.gDW^−1^.h^−1^)
Triple	ADHEr, LDH_D, PTAr	OptKnock [33], FastKnock	0.08	9.37	0.94	0.75	0.79	-**9.36 (uptake)**
	ADHEr, LDH_D, PFL	OptKnock, FastKnock (best production rate)	0.12	**12.24**	**1.22**	**1.46**	1.53	−5.87 (uptake)
	PTAr, PYK, GLCpts	OptKnock, FastKnock	0.09	9.32	0.93	0.83	0.87	3.24 (production)
	PFL, LDH_D, GLCpts	Experimental [71] (production is lower than considered threshold)	**0.16**	0.71	0.07	0.11	0.11	16.78 (production)
	ADHEr, ATPS4r, LDH_D	FastKnock (best SoGC)	**0.16**	8.90	0.89	1.42	**3.01**	−8.76 (uptake)
Quadruple	PTAr, PYK, ATPS4r, SUCD1i	OptKnock	**0.16**	1.18	0.11	0.18	0.01	9.03 (production)
	ADHEr, LDH_D, PFL, THD2	FastKnock (best production rate)	0.11	**12.72**	**1.27**	**1.39**	2.85	−6.12 (uptake)
	ADHEr, LDH_D, HEX1, THD2	FastKnock (best SoGC)	0.13	9.88	0.98	1.28	**3.09**	−**8.77 (uptake)**
Quintuple	ADHEr, LDH_D, PTAr, PYK, GLCpts	OptKnock, FastKnock	0.05	9.96	0.99	0.49	1.19	−**9.51 (uptake)**
	ADHEr, LDH_D, PFL, ACKr, FORt	Experimental [71], FastKnock	0.08	9.57	0.95	0.76	0.80	−9.16 (uptake)
	ADHEr, LDH_D, HEX1, THD2, DRPA	FastKnock (best SoGC)	**0.13**	9.87	0.98	**1.28**	**3.10**	−8.76 (uptake)
	ADHEr, LDH_D, GLUDy, PFL, THD2	FastKnock (best production rate)	0.10	**12.77**	**1.27**	1.27	2.61	−6.17 (uptake)

**Table 6:** MCSEnumerator results for ethanol production in the iAF1260 that are lethal for the microorganism.

Solution	Deleted reactions				Growth rate (h^−1^)	Ethanol production rate (mmol.gDW^−1^.h^−1^)
1	ACKr	EDA	PGI	TKT1	0.0	20.88
2	ATPS4rpp	EDA	PGI	TKT1	0.0	31.45
3	EDA	PGI	PTAr	TKT1	0.0	20.88

## Data Availability

All data generated or analyzed during this study are included in this published article [and its supplementary information files]. Our implementation of the FastKnock method in Python is publicly available at https://github.com/leilahsn/FastKnock.
